# Zika virus propagation and release in human fetal astrocytes can be suppressed by neutral sphingomyelinase-2 inhibitor GW4869

**DOI:** 10.1038/s41421-018-0017-2

**Published:** 2018-04-24

**Authors:** Yunlong Huang, Yuju Li, Hainan Zhang, Runze Zhao, Ran Jing, Yinghua Xu, Miao He, Justin Peer, Yeong C. Kim, Jiangtao Luo, Zenghan Tong, Jialin Zheng

**Affiliations:** 1 Center for Translational Neurodegeneration and Regenerative Therapy, Shanghai Tenth People’s Hospital Affiliated to Tongji University School of Medicine, Shanghai, China; 2 Department of Pharmacology & Experimental Neuroscience, University of Nebraska Medical Center, Omaha, NE 68198 USA; 3 Department of Pathology and Microbiology, University of Nebraska Medical Center, Omaha, NE 68198 USA; 4 Department of Genetics, Cell Biology and Anatomy, College of Medicine, University of Nebraska Medical Center, Omaha, NE 68198 USA; 5 Department of Biostatistics, College of Public Health, University of Nebraska Medical Center, Omaha, NE 68198 USA

## Abstract

Zika virus (ZIKV) is a neurotrophic flavivirus that is capable of infecting humans, leading to brain abnormalities during fetal development. The ZIKV infectivity in neural target cells remains poorly understood. Here, we found that ZIKV specifically infected glial fibrillary acidic protein- and S100B-positive primary human astrocytes derived from fetal brains. In contrast, neuron-specific Class III β-tubulin (TuJ1)-positive neurons in the astrocyte cultures and SOX2-positive neural progenitor cells derived from the fetal brains were less susceptible to ZIKV infection compared with astrocytes. The infected astrocytes released competent viral particles and manifested programmed cell death with a progressive cytopathic effect. Interestingly, ZIKV infection in human fetal astrocytes induced a significant increase of extracellular vesicles (EVs). Treatment with GW4869, a specific inhibitor of neutral sphingomyelinase-2, decreased EV levels, suppressed ZIKV propagation, and reduced the release of infectious virions in astrocytes. Therefore, ZIKV infects primary human fetal astrocytes and the infection can be suppressed by neutral sphingomyelinase-2 inhibitor GW4869. Further investigation into sphingomyelin metabolism and EVs may provide insights to the therapeutic treatment of ZIKV infection.

## Introduction

Zika virus (ZIKV) is a single-stranded RNA virus of the Flaviviridae family^1^. It is transmitted to humans primarily through the bites of infected *Aedes* mosquitoes, though both perinatal/in utero and sexual transmission have been reported^2–4^. Initially discovered in 1947, ZIKV infection has been reported in Americans since 2014, with a major outbreak in Brazil starting in 2015. Genetic studies have revealed that the ZIKV has three distinct genotypes: West African (Nigerian cluster), East African (MR766 prototype cluster), and Asian^5^. It has been postulated that the virus evolved from the Asian genotype and spread to French Polynesia (2013) then to Brazil (2015)^5^. Infection of ZIKV has been suggested to cause neuropathologies such as microcephalic fetuses^6, 7^. Moreover, ZIKV infection might also be associated with an increased incidence of Guillain–Barre Syndrome in adults^8^. The mechanisms for those neuropathologies are not clear. In the case of microcephaly, recent studies in humans have shown that Zika viral antigens were only found in neurons and glia cells with no immunohistochemical evidence of infection in other vital tissues^9^, which suggests a neurotropism of ZIKV that evades immune control.

The molecular basis for ZIKV replication in the cells of neural lineage remains an intense area of study. Recent structural studies revealed that ZIKV has a similar structure to other flaviviruses^10, 11^. For flavivirus, infection typically initiates through clathrin-mediated endocytosis, which is followed by removal of the envelope, disruption of the nucleocapsid, and release of the viral genome into the cytoplasm^12, 13^. It has been proposed that the entry receptor tyrosine-protein kinase receptor UFO (AXL) is the target for the envelope protein; AXL is highly expressed by human radial glial cells, astrocytes, endothelial cells, and microglia in the developing human cortex, progenitor cells in developing retina, and human stem cell-derived cerebral organoids^14^. Data from mouse models demonstrate that ZIKV can directly infect different lineages of mouse neural progenitor cells (NPCs) and immature neurons in vivo, leading to an impaired NPC development and microcephaly-like pathology^15^. More recently, vertical transmission of ZIKV has been reported and shown to have detrimental effects on cortical neural progenitors of offspring animals in vivo^16, 17^. Astrocytes are important glia cells that are critical for both the proper development and health of the central nervous system (CNS). Despite their importance, little is known for their role in ZIKV viral replication and pathogenesis.

Extracellular vesicles (EVs), which include microvesicles and exosomes, have emerged as an important factor in cell-to-cell communication^18, 19^. EVs range in size from 40 nm to 1 μm and are shed either by the budding of plasma membranes or exocytosis from multivesicular bodies, delivering cytokines, nucleic acids, lipids, and proteins to target cells^20–25^. EVs are typically generated through endosomal sorting complexes required for transport (ESCRT)-mediated process or through the formation of ceramide from sphingomyelin by sphingomyelinase^26, 27^. In pathological conditions, EVs have been proposed as biomarkers for viral infection and for various neurological disorders. Specifically, viral infections are known to manipulate EV pathways and viral proteins are found within EVs, both of which could support viral infection and evade host immune response as explained by the “Trojan horses” hypothesis (see recent reviews at refs. ^28, 29^). However, the role of EVs and the upstream ceramide pathway in ZIKV infection remain unknown.

Although there is a significant effort to identify viral suppression strategies for ZIKV^30^, currently no vaccines or specific therapies are available to treat ZIKV infection. In this study, we first investigated the effect of ZIKV infection in a unique human fetal astrocyte culture. We demonstrated that primary human fetal astrocytes are more susceptible to ZIKV compared with neurons in the cultures or NPCs derived from the same fetal tissues. Interestingly, GW4869, a neutral sphingomyelinase-2 (nSMase2) inhibitor, effectively decreased EV levels and inhibited ZIKV propagation in human astrocytes.

## Results

### ZIKV establishes productive infection in primary human fetal astrocytes

**Fig. 1:**
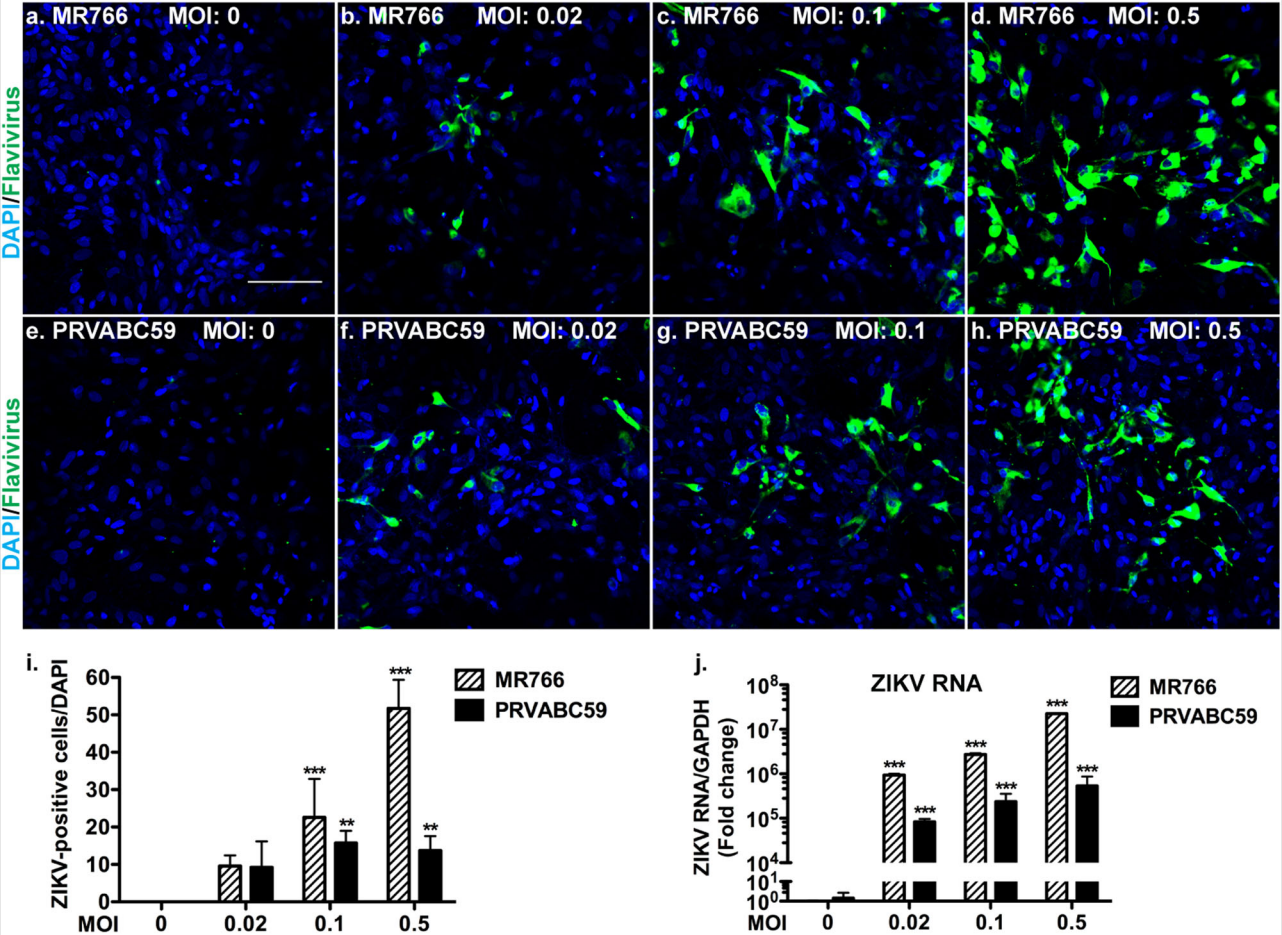
Efficient infection of astrocytes by ZIKV. Human fetal astrocytes were infected with ZIKV strains MR766 or PRVABC59 at the indicated MOI for 24 h, washed, then cultured for another 24 h. Supernatant from uninfected Vero cells was used for mock-infected control. **a**–**h** At the experimental endpoint, ICC of flavivirus antigen (green color) was performed. DAPI (blue color) was used as a nuclear counterstain. Panels are representative of three separate donors. Scale bar: 100 µm. Images were acquired through a Zeiss LSM 710 confocal microscope. **i** ZIKV-positive cells in **a**–**h** were quantified and shown as percentage of total cells in the culture. **j** RNA was isolated and expression ZIKV RNA was determined through real-time RT-PCR. Data were normalized to GAPDH and presented as fold change compared to mock-infected control. **p < 0.001; ***p < 0.0001 as compared to mock-infected control

We obtained ZIKV strains MR766 and PRVABC59 through a commercial source (ZeptoMetrix Corp. Buffalo, NY) and propagated the viral strains in a Vero cell line as previously described^31^. Among these viral strains, MR766, an African strain, has been demonstrated to infect neural stem cells (NSCs) and lead to NSC cell death^31–33^. PRVABC59 was isolated in Puerto Rico and is genetically close to the circulating viral strains in Brazil (GeneBank Accession: KU501215). Viral titers were determined by counting plaque-forming units (PFU) in viral plaque-forming assays (PFA) in Vero cells (Supplementary Figure S1). To prepare primary human fetal astrocytes for ZIKV infection, we obtained single-cell suspensions dissociated from fetal (12–16 weeks) brain tissues, which consisted of mostly progenitor cells. We differentiated the cells to astrocytes in serum medium and more than 95% of the cells were positive for GFAP as we previously described^34–36^. The viral stock was then used to infect human fetal astrocytes with the indicated multiplicity of infection (MOI). After infection for 24 h, remaining Zika virions in the supernatant were removed. Infected astrocytes were washed with fresh media and cultured for another 24 h. At the experimental endpoint, astrocytes were fixed with 4% paraformaldehyde in phosphate buffered saline (PBS) and stained with antibodies against flavivirus antigen that also cross-react with ZIKV through immunocytochemistry (ICC) to confirm the presence of the virus (Fig. 1a–h). Notably, both MR766 and PRVABC59 were capable of infecting fetal human astrocytes. PRVABC59 appeared to have lower infection efficiency and viral RNA compared with MR766 (Fig. 1i and j), suggesting that although there is general cellular tropism and susceptibility of astrocytes to ZIKV, viral genetics may also affect the replication kinetics.

To determine whether ZIKV infection of astrocytes is a productive infection, we performed experiments as outlined in Supplementary Figure S2a. Briefly, at 24-h post ZIKV infection, astrocytes were washed three times with PBS, and incubated for an additional 24 h. Both PBS from last wash and astrocyte medium from the infected cultures were collected and added to Vero cells for the detection of any infectious Zika virions. Vero cells incubated with the last wash of MR766-infected astrocytes generated minimal levels of infection (Supplementary Figure S2b). In contrast, Vero cells incubated with supernatants from MR766-infected astrocytes generated robust ZIKV infection (Supplementary Figure S2c). Similarly, Vero cells incubated with the last wash of PRVABC59-infected astrocytes generated minimal levels of infection (Supplementary Figure S2d) but supernatants from PRVABC59-infected astrocytes generated robust ZIKV infection (Supplementary Figure S2e), suggesting infected astrocyte cultures produced and released many ZIKV virions into the supernatant. To quantitatively determine the viral replication and virion production, we used real time RT-PCR and PFA to measure ZIKV RNA and virions, respectively, during and after infection in astrocytes (Supplementary Figure S3a). ZIKV RNA increased exponentially at 48-h post infection compared to samples taken immediately after infection or the 2-h post infection time point, suggesting a significant viral replication (Supplementary Figure S3b). Similarly, ZIKV virions increased at 48-h post infection compared with the virion level during infection (Supplementary Figure S3c). Together, these results suggest that ZIKV infection of astrocytes is a productive infection.

### ZIKV specifically infects GFAP- and S100B-positive human fetal astrocytes

**Fig. 2:**
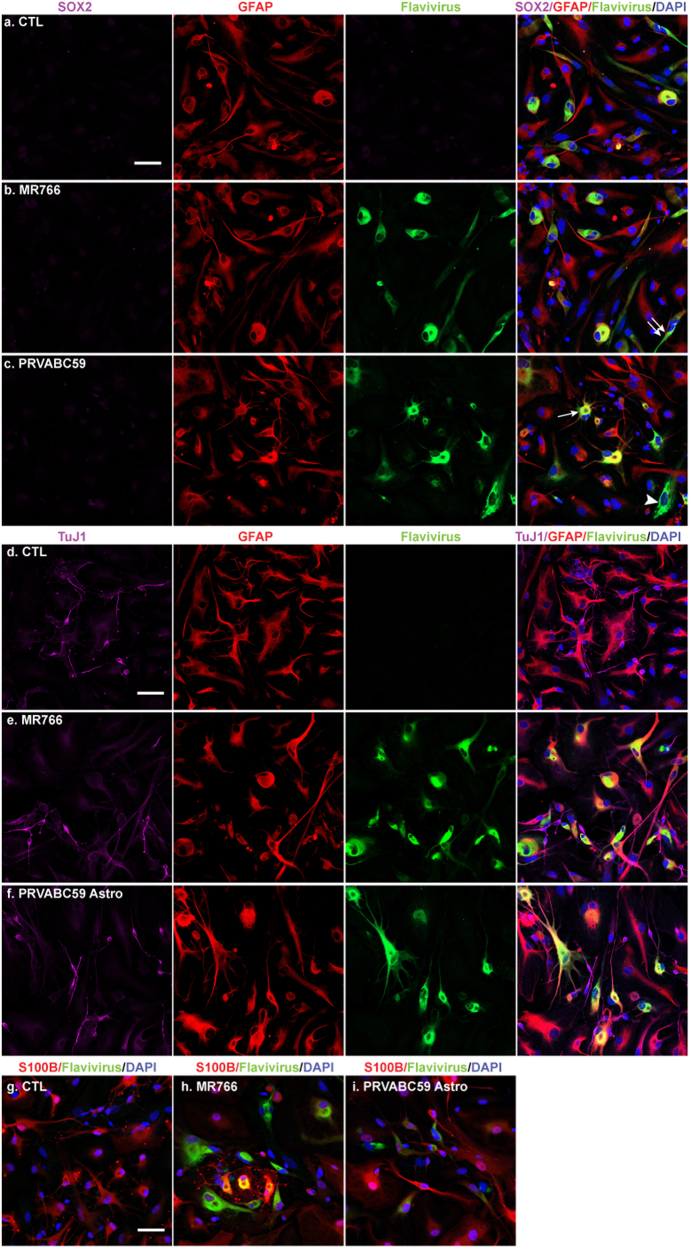
Characterization of ZIKV-infected cells in the fetal astrocyte cultures. Human fetal astrocytes were mock-infected or infected with ZIKV strains MR766 or PRVABC59 at the MOI of 0.5 for 24 h, washed, then cultured for another 24 h. **a**–**c** At the experimental endpoint, ICC of SOX2 (purple color), GFAP (red color), and flavivirus antigen (green color) was performed. The white arrow, double arrow, and arrowhead denotes bipolar-shaped GFAP-positive, and fibrous GFAP-positive, and GFAP-negative ZIKV-infected cells, respectively. **d**–**f** ICC of TuJ1 (purple color), GFAP (red color), and flavivirus antigen (green color) was performed. **g**–**i** ICC of S100B (red color) and flavivirus antigen (green color) was performed. DAPI (blue color) was used as a nuclear counterstain for all ICC imaging. Panels are representative of three separate donors. Images were acquired through a Zeiss LSM 710 confocal microscope. Scale bar: 50 μm. CTL control

Next, we investigated the morphological changes of human astrocytes upon ZIKV infection. Astrocytes are classified as highly ramified protoplasmic astrocytes in gray matter and fibrous astrocytes in white matter^37^. A great majority (>80%) of our cultured astrocytes were positive for GFAP (Fig. 2a–f). The percentage of GFAP-positive cells can be enhanced to more than 95% by using another anti-GFAP antibody (Abcam, Ab4674, data not shown). In uninfected cultures, astrocytes were negative for flavivirus antigen staining (Fig. 2a). The anti-flavivirus antibody (4G2) binds to a conserved epitope on the E protein and has been shown to recognize several viruses including ZIKV in the flavivirus family^38^. Upon infection of ZIKV strain MR766 (Fig. 2b) or PRVABC59 (Fig. 2c), the flavivirus antigen was co-localized to astrocytes with diverse sizes and morphologies, including bipolar-shaped (Fig. 2c, white arrow) and fibrous (Fig. 2b, white double arrow) morphologies. Interestingly, a small percentage (<10%) of GFAP-negative cells in the cultures were apparently infected by the ZIKV (Fig. 2c, white arrowhead).

Although a great majority of cells infected by ZIKV were GFAP positive in our astrocyte cultures, recent identification of cortical neural progenitors/radial glia cells as primary targets of ZIKV^17, 31^, which are also GFAP positive, raises the question whether there were any residue NPCs remaining in the fetal astrocyte cultures. We have checked Sox2 (NPC marker) staining in our astrocyte cultures, using human NPC cultures derived from fetal brain tissues as positive controls^39–41^. In uninfected cultures, a majority of NPCs were positive for SOX2 and negative for flavivirus antigen staining (Supplementary Figure S4a). We found no positive staining of Sox2 in the astrocyte cultures (Fig. 2a–c). Interestingly, when human NPC cultures were compared with astrocytes side-by-side in terms of ZIKV infection, both MR766 and PRVABC59 strains of ZIKV were found to poorly infect NPCs (Supplementary Figure S4b and c). In contrast, the same titer of ZIKV established effective infection in astrocyte (Fig. 2b and c). Likewise, we checked Iba1 (microglia marker) staining in the astrocyte cultures using human microglia^42^ as positive controls and found no staining of Iba1 (data not shown). In addition, ICC on the cultures with antibodies against endothelial cell marker CD31, oligodendrocyte progenitor cells marker NG2, and oligodendrocyte marker myelin-PLP revealed little positive staining (data not shown). The astrocyte cultures are known to contain a small number of neurons^43^. To check whether these cells are infected during ZIKV infection, we stained them with neuron-specific class III β-tubulin (TuJ1) along with flavivirus antigen. In uninfected cultures, neurons were negative for flavivirus antigen staining (Fig. 2d). Upon infection of MR766 (Fig. 2e) or PRVABC59 (Fig. 2f), the flavivirus antigen was co-localized to astrocytes but not TuJ1-positive neurons.

Consistent with the immunostaining data, gene expression analysis showed that the astrocytes expressed higher levels of GFAP, Glutamate Aspartate Transporter (GLAST)/Excitatory Amino Acid Transporter 1 (EAAT1), Excitatory amino acid transporter 2 (EAAT2) (Supplementary Figure S5a–c), compared with those of Nestin- and SOX2-expressing NPC or CX3CR1-expressing microglia (Supplementary Figure S5d–f). EAAT1/GLAST and EAAT2 are both glutamate transporters that are highly expressed in astrocytes. Therefore, it is unlikely that there are any NPC or microglia in our fetal astrocyte cultures and ZIKV infection appears to be very specific for astrocytes in the fetal astrocyte cultures.

To further confirm that the infected cells are indeed astrocytes, we double labeled astrocytes with antibodies against flavivirus antigen and S100 calcium-binding protein B (S100B). S100B is a member of the S100 family of EF-band calcium-binding proteins and a commonly used astrocyte marker only expressed by a subtype of mature astrocytes^44, 45^. As expected, S100B was expressed in astrocyte cultures to varying degrees (Fig. 2g). Upon MR766 (Fig. 2h) and PRVABC59 (Fig. 2i) strains of ZIKV infection, the flavivirus antigen was localized to both S100B-positive and -negative cells in the cultures, indicating ZIKV infects astrocytes of different maturation stages.

### ZIKV infection in astrocytes causes a cytopathic effect

**Fig. 3:**
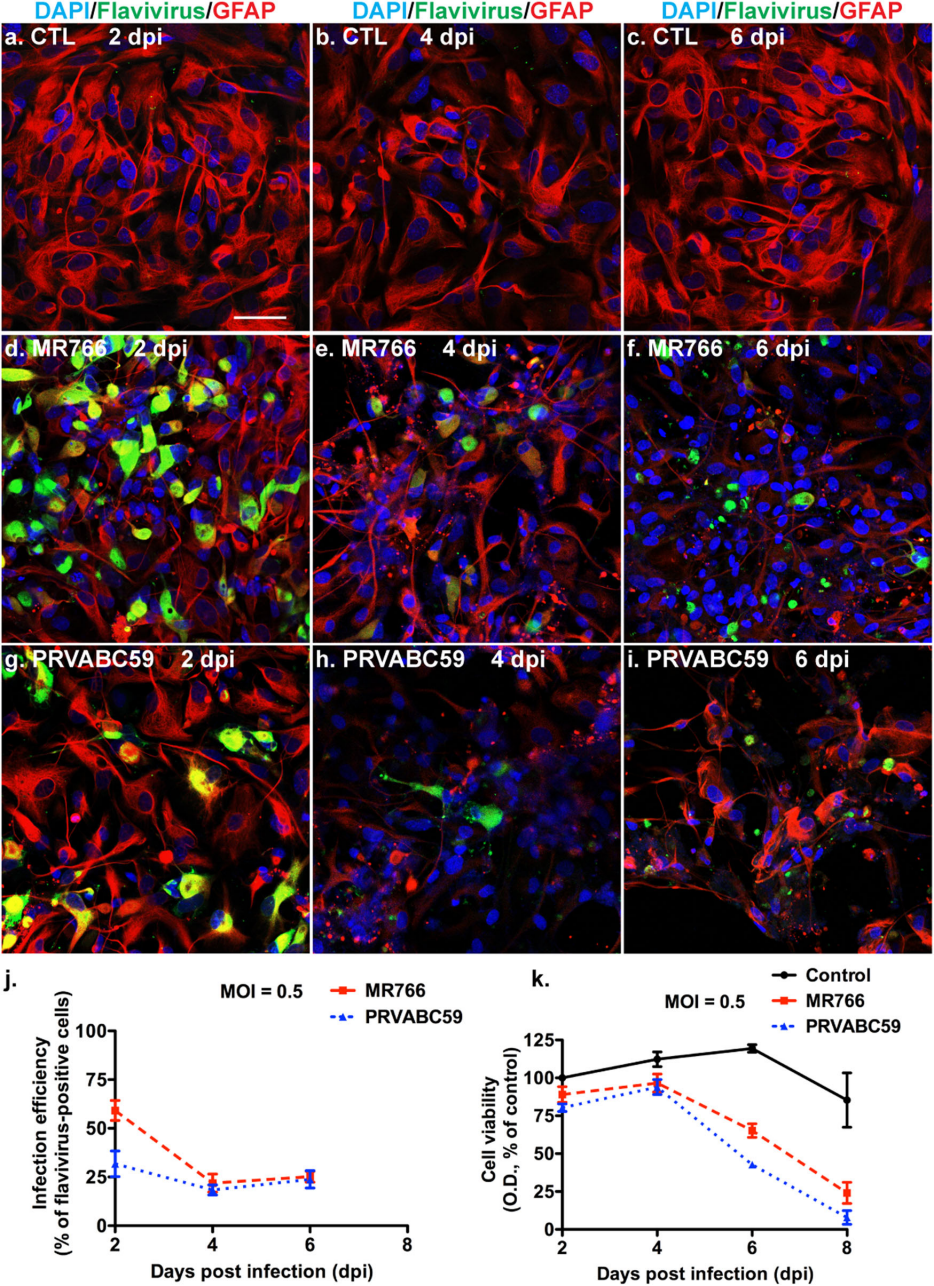
ZKV induces cytopathic effects in astrocytes. Human fetal astrocytes were infected with ZIKV strains MR766 or PRVABC59 at the MOI of 0.5 for the indicated length of time. **a**–**i** ICC of flavivirus antigen (green color) and GFAP (red color) was performed. DAPI (blue color) was used as a nuclear counterstain. Scale bar: 50 μm. **j** ZIKV-positive cells in **a**–**l** were quantified and shown as percentage of total cells in the culture. **k** Cell viability of the astrocyte cultures were determined by the CellTiter 96^®^ AQueous One Solution Assay at every other day for 8 days. Results were normalized as percentage of mock-infected control at 2-day post infection. CTL control. Representative of three independent experiments was shown

MR766 strain of ZIKV has been demonstrated to infect human NSCs and lead to NSC cell death^31–33^. To determine whether ZIKV infection leads to similar cell death in astrocytes, we infected primary human fetal astrocytes with MR766 or PRVABC59 and observed cell viability for 8 days. MR766 and PRVABC59 had 59% and 29% infection efficiency, respectively, at 2-day post infection (Fig. 3a–j). The highest infection levels were shown at this time point, and infection efficiencies for both viral strains dropped to around 20% at the subsequent time points. The infection also caused a progressive cytopathic effect for both strains; pyknotic nuclei were present at infected cultures at 4- and 6-day post infection, whereas they were only rarely visualized in uninfected cultures (Fig. 3a–j). At 8-day post infection, the cytopathicity in the infected cultures was rampant and very few cells were left for ICC (data not shown). Consistent with the cellular morphology, both MR766 and PRVABC59 significantly affected astrocyte viability at 6- and 8-day post infection (Fig. 3k). Together, the dynamics of peaked ZIKV infection and the subsequent cell death suggest that ZIKV infection may have a cause-and-effect relationship with cell death in fetal astrocytes.

**Fig. 4:**
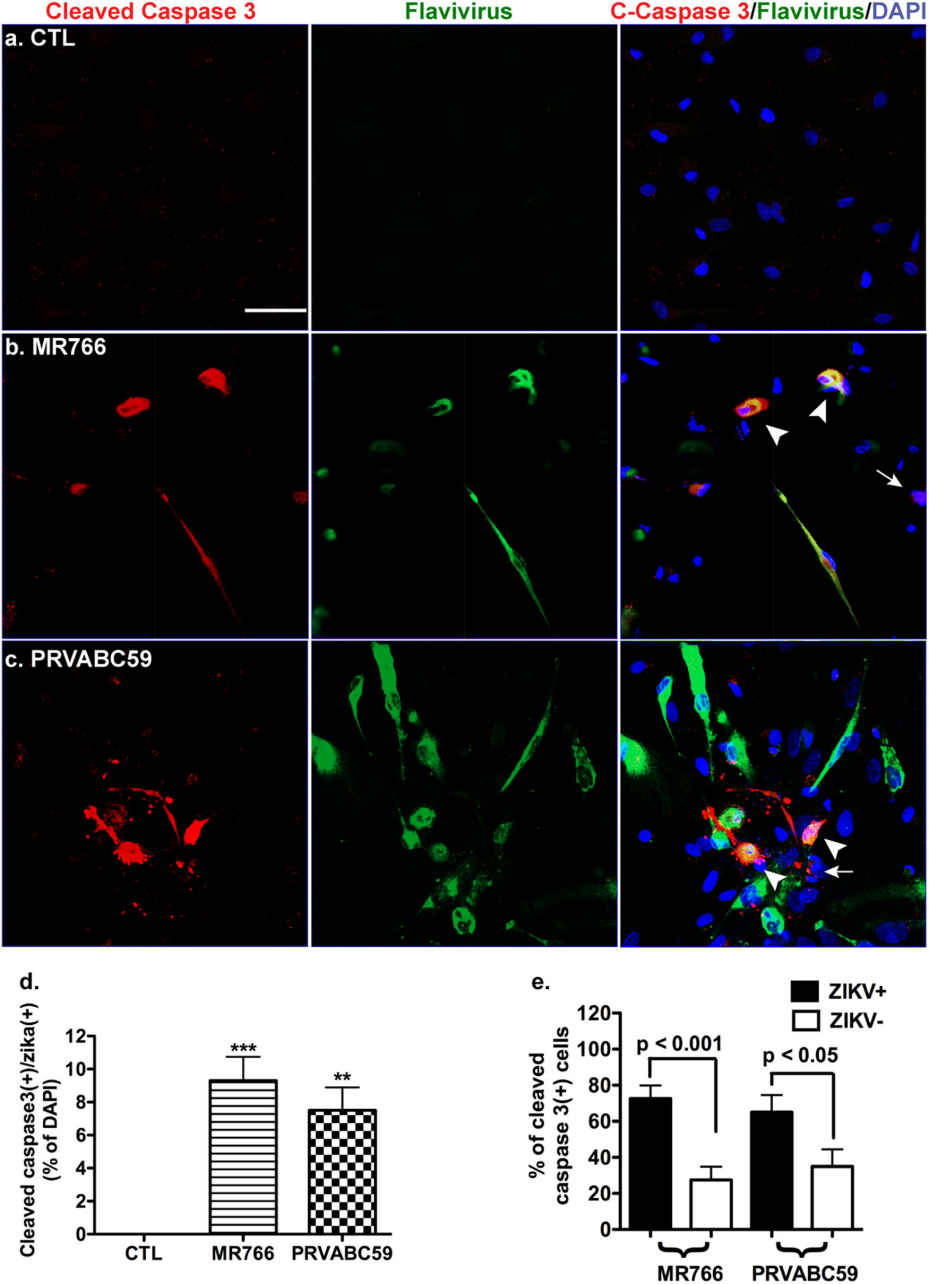
ZKV induces apoptosis in astrocytes. Human fetal astrocytes were infected with ZIKV strains MR766 or PRVABC59 at the MOI of 0.5 for 24 h, washed, then cultured for another 24 h. **a**–**c** ICC of activated caspase 3 (red color) and flavivirus antigen (green color) and was performed. DAPI (blue color) was used as a nuclear counterstain. The white arrowhead and arrow denotes apoptotic infected astrocyte and apoptotic uninfected astrocyte, respectively. Scale bar: 50 μm. **d** ZIKV- and activated caspase 3-dual-positive cells in **a**–**I** were quantified and shown as percentage of total cells in the cultures. Ten random fields from each group were manually counted by a blinded assessor. CTL control. **e** Cells positive with activated caspase 3 were broken down between ZIKV-positive and ZIKV-negative groups. Results are normalized as percentage of total activated caspase 3-positive cells and are representative of three independent experiments. **p < 0.01, ***p < 0.001, in comparison to mock-infected control (ANOVA, *N* = 10)

Using ICC, we found that ZIKV-infected cultures had significantly higher levels of cells that were positive for activated caspase 3 (Fig. 4a–d) compared with mock-infected cultures. The elevated levels of apoptosis were found both in the MR766 strain- and PRVABC59 strain-infected cells (Fig. 4d). After quantification, we found that 72.5 ± 7.3% and 65.0 ± 9.5% of activated caspase 3-positive cells were ZIKV infected. In contrast, 27.5 ± 7.3 and 35 ± 9.5% of activated caspase 3-positive cells were uninfected (Fig. 4e). Together, these data indicate that the mechanism of ZIKV-induced cell death is through apoptotic pathway and the death of uninfected cells is likely a bystander cell death.

### ZIKV infection alters EV levels in human fetal astrocyte cultures

**Fig. 5:**
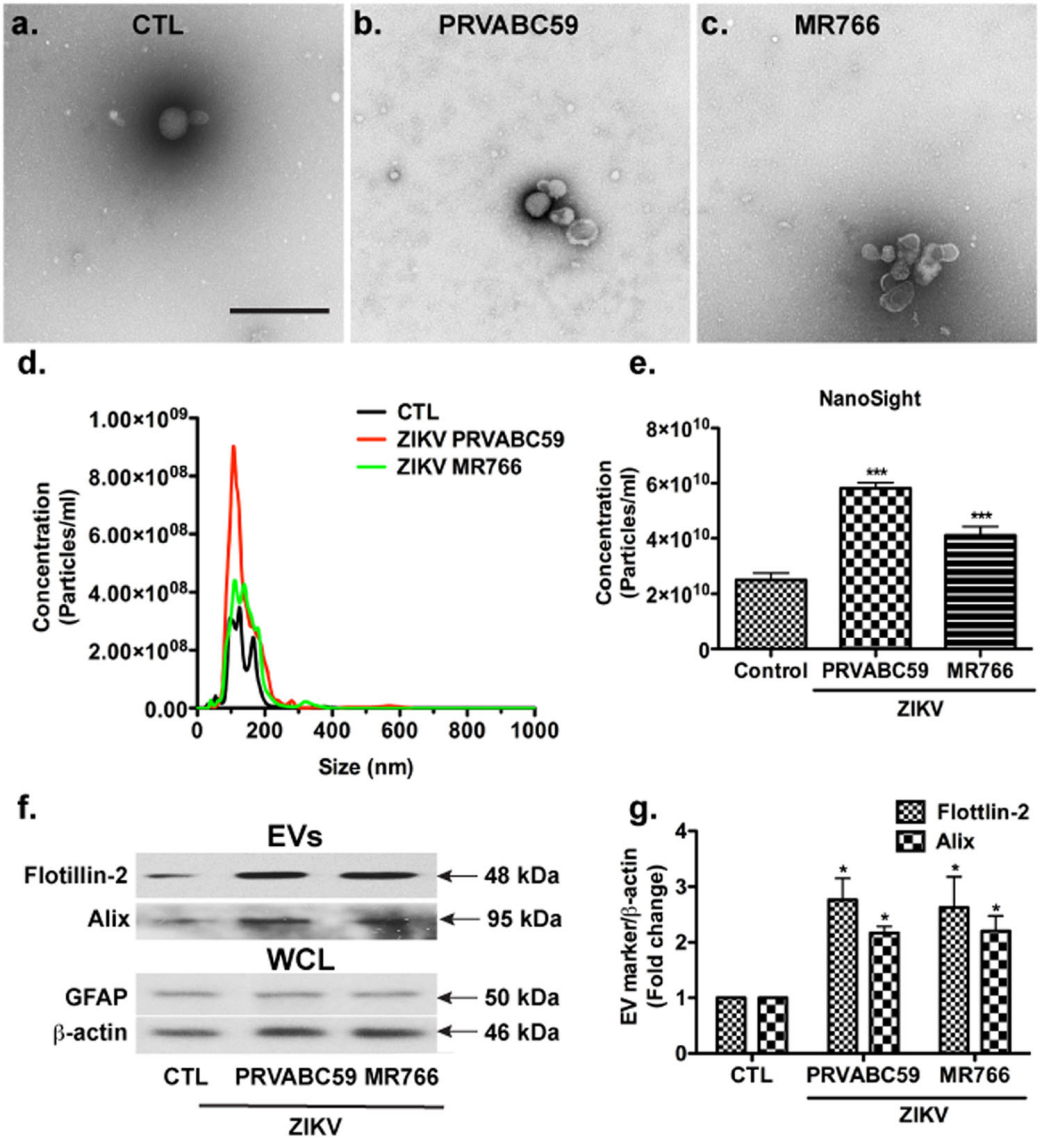
ZIKV infection increases EV release from human astrocytes. Astrocytes were infected with ZIKV stains PRVABC59 or MR766 for 24 h and the cultures were washed and supplemented with fresh media for another 24 h. **a**–**c** Electron micrograph of EVs from the mock-infected control and ZIKV strains MR766- or PRVABC59-infected astrocytes. Scale bar: 500 nm. **d** EVs were isolated from normalized volumes of supernatants in mock-infected and ZIKV-infected astrocyte cultures based on whole cell protein concentrations. EVs were visualized through NanoSight for number of EVs (1:100, *y*-axis) and size of EVs (nm, *x*-axis). **e** Quantifications of EV number were performed through NanoSight. Results are representative of three independent experiments. ***p < 0.0001 in comparison to mock-infected control (ANOVA, *N* = 5). **f** The levels of flotillin-2 and Alix in EVs, as well as the levels of GFAP and β-actin in WCL were determined by Western blot. **g** Densitometric quantifications of flotillin-2 and Alix in EVs were presented as a ratio to β-actin and normalized as fold changes to mock-infected control. *p < 0.05 in comparison to mock-infected control (ANOVA, *N* = 3). CTL control

After characterization of ZIKV infection in astrocytes, we investigated ZIKV infection processes that may be targeted for drug development. EVs and viruses may cross paths in biogenesis^46^, therefore, investigation on EVs and the upstream ceramide pathway during this pathological condition may provide insight to ZIKV propagation and release. We isolated EVs from culture supernatants through the differentiation centrifugation method as we previously described^47^. Our EV preparation from astrocytes-conditioned medium was enriched with EVs 50–200 nm in diameter as determined by transmission electron microscopy (TEM, Fig. 5a–c). The EVs from mock-infected controls and ZIKV-infected astrocytes were subjected to Nanoparticle Tracking Analysis (NTA, NanoSight NS300, Malvern Instruments Inc., Westborough MA). Consistent with TEM data, EVs isolated from mock-infected astrocytes had a mean particle size of 133 nm ± 47 nm in NanoSight, whereas EVs isolated from PRVABC59 and MR766-infected astrocytes had mean particle sizes of 138 nm ± 61 nm and 142 nm ± 48.7 nm, respectively (Fig. 5d), which suggested that ZIKV infection does not change the size of EVs in astrocytes significantly. In contrast, PRVABC59 or MR766 infection significantly increased the number of EVs released from astrocytes (Fig. 5d and e). To validate the increase of EVs, we used Western blotting to determine the presence of the EV markers Flotillin-2 and Alix in the protein lysates derived from the EV pellets (Fig. 5f). To ensure that EVs were isolated from the same amount of cells, we also determined GFAP and β-actin levels in whole cell lysates (WCL). Both GFAP and β-actin levels in infected cells were comparable to those of the mock-infected control (Fig. 5f). Quantification of Flotillin-2 in EVs against β-actin in WCL identified that ZIKV infection dramatically increased the levels of Flotillin-2 and Alix in the EV lysates, confirming that ZIKV infection increases EV biogenesis in astrocytes (Fig. 5g). As a control experiment, we also determined levels of ZIKV RNA in EVs compared with those from an equivalent volume of supernatant prior to and after EV isolation. The level of ZIKV RNA in EVs accounts for 25% of ZIKV RNA from an equivalent volume of supernatant prior to EV isolation and for 50% of ZIKV RNA after EV isolation, suggesting that although a majority of ZIKV RNA remained in supernatant after EV isolation, around 25% of ZIKV RNA did make it to the EVs (Supplementary Figure S6).

### Inhibition of neutral sphingomyelinase-2 by GW4869 halts ZIKV infection in astrocytes

**Fig. 6:**
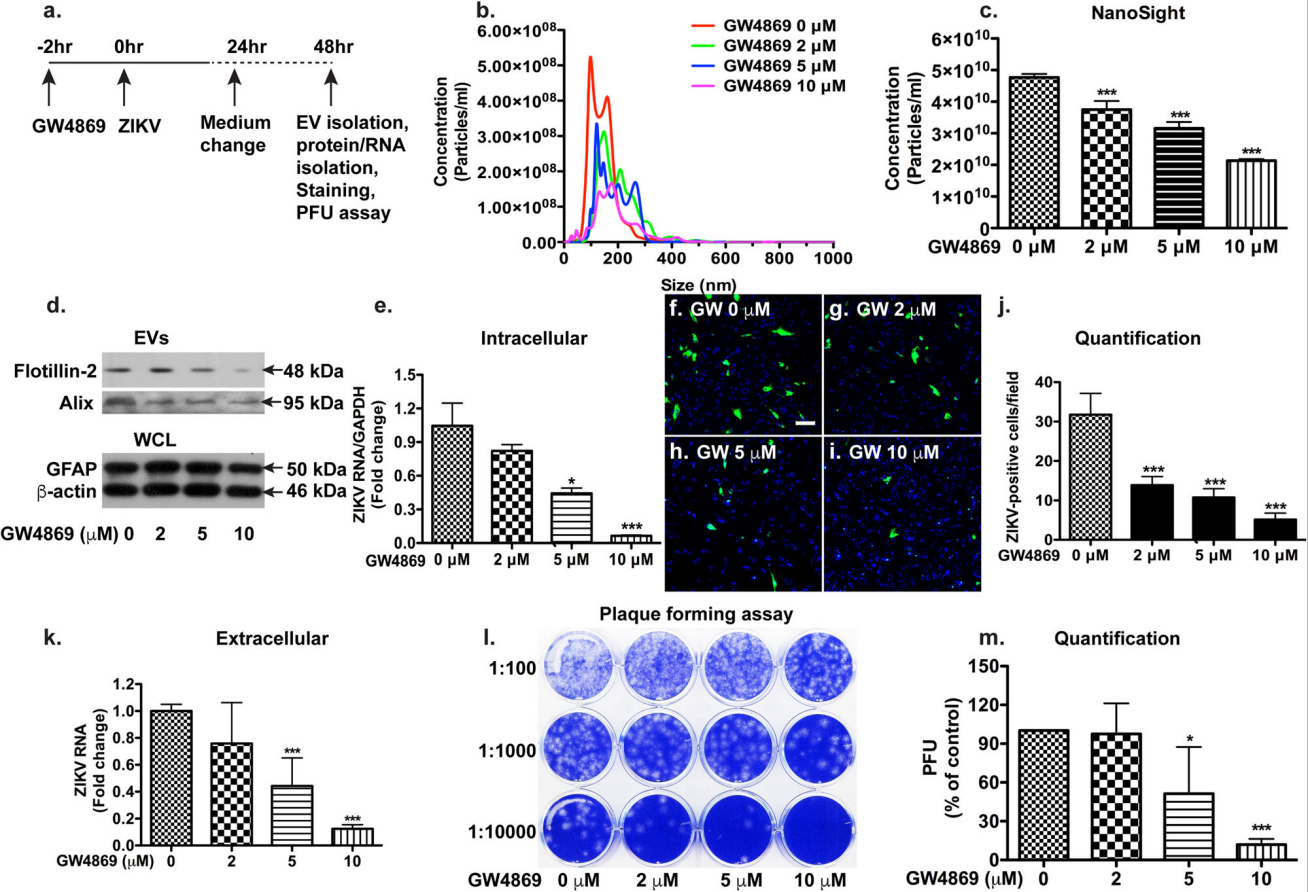
GW4869 inhibits PRVABC59 strain of ZIKV infection in human astrocytes. Astrocytes were infected with ZIKV stains PRVABC59 and treated with doses of GW4869 ranging from 2 μM to 10 μM. After 24 h, the cultures were washed and treated with same doses of GW4869 in fresh medium for another 24 h. **a** Experimental scheme. **b**–**d** EVs were isolated from culture supernatants and visualized through NanoSight (**b**). Quantifications of NanoSight data. ***p < 0.0001 in comparison to ZIKV group (ANOVA, *n* = 5) (**c**). The levels of flotillin-2 and Alix in EVs, as well as the levels of GFAP and β-actin in WCL were determined by Western blot (**d**). **e** ZIKA RNA was detected in total cellular RNA through real-time RT-PCR. Data were normalized to GAPDH and presented as fold change compared to ZIKV group. **f**–**i** Immunocytochemistry of flavivirus antigen was performed on the ZIKV-infected astrocytes. DAPI was used as a nuclear counterstain. Results are representative of three independent experiments. Scale bar: 50 μm. **j** Quantification of immunofluorescence data was performed with counting flavivirus antigen in infected cells. **k** ZIKA RNA was detected in total RNA isolated from cell-free supernatants through real-time RT-PCR. **l**,** m** After GW4869 treatment, cell-free culture supernatants were added to Vero cells and overlaid with Agar gel. Viral PFU was determined at 4-day post inoculation through crystal violet staining (**l**). Viral plaques were manually counted and calculated as PFU/ml (**m**). *p < 0.05; ***p < 0. 001, as compared to the ZIKV group

**Fig. 7:**
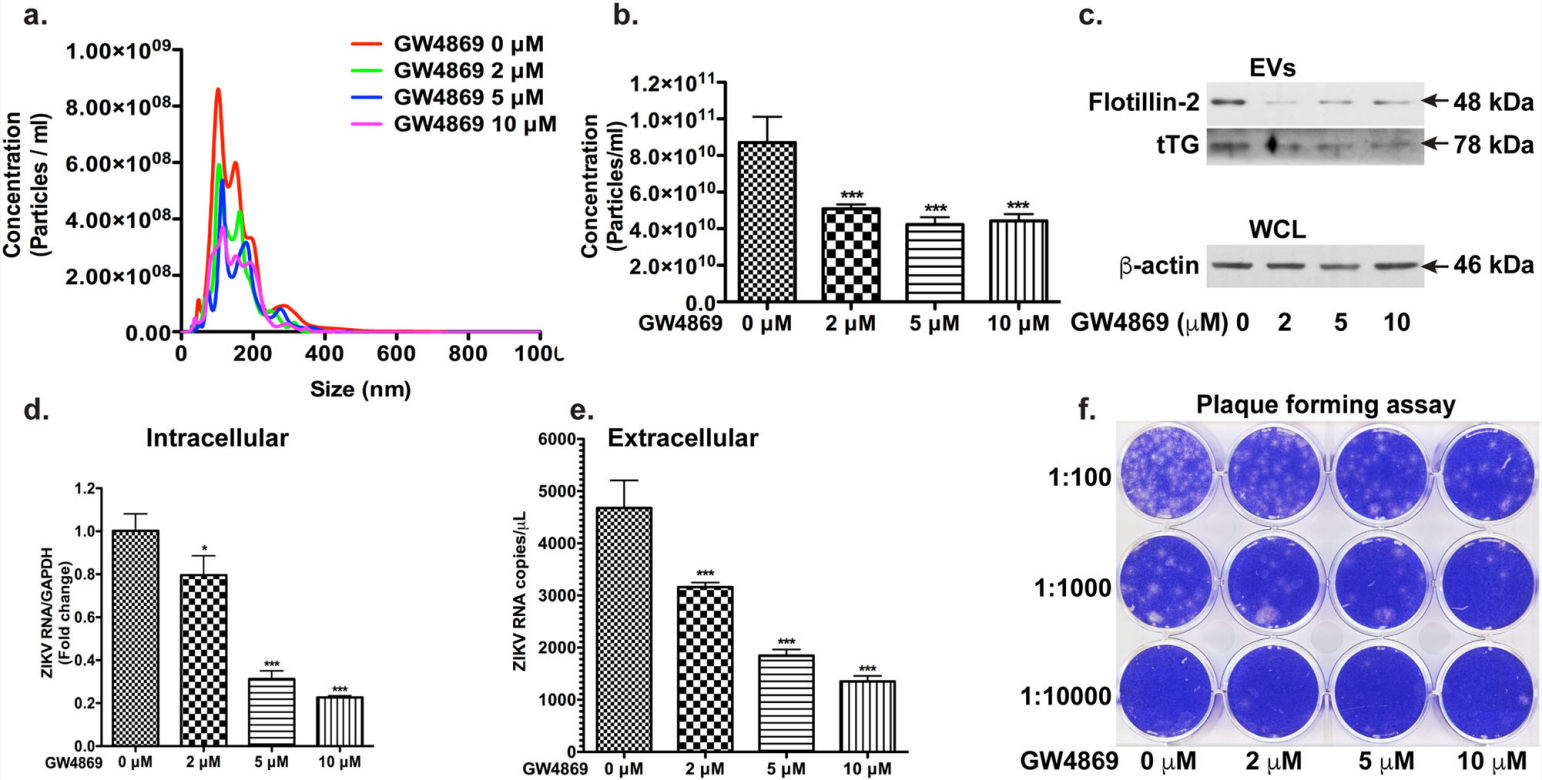
GW4869 inhibits MR766 strain of ZIKV infection in human astrocytes. Astrocytes were infected with ZIKV stains MR766 and treated with doses of GW4869 ranging from 2 μM to 10 μM. After 24 h, the cultures were washed and treated with same doses of GW4869 in fresh medium for another 24 h. **a**–**c** EVs were isolated from culture supernatants and visualized through NanoSight (**a**). Quantifications of NanoSight data. ***p < 0.0001 in comparison to ZIKV group (ANOVA, *n* = 5) (**b**). The levels of flotillin-2 and tTG in EVs, as well as the levels of β-actin in WCL were determined by Western blot (**c**). **d** ZIKA RNA was detected in total cellular RNA through real-time RT-PCR. Data were normalized to GAPDH and presented as fold change compared to ZIKV group. **e** ZIKA RNA was detected in total RNA isolated from cell-free supernatants through real-time RT-PCR. Quantitative Genomic RNA from ZIKV (ATCC) was used as standard for viral copy determination. **f** After GW4869 treatment, cell-free culture supernatants were added to Vero cells and overlaid with Agar gel. Viral PFU were determined at 4-day post inoculation through crystal violet staining

To further determine the effects of EVs and the upstream ceramide pathway on ZIKV infection, we used GW4869, which is known to inhibit nSMase-2 and decrease EV levels^27, 48^, in the ZIKV PRVABC59 strain-infected astrocytes (Fig. 6a). GW4869 is known to have little toxicity to either cultured cells or animals^49^. We have tested and found no toxicity of GW4869 to the infected astrocytes (Supplementary Figure S7). Treatment with GW4869 reduced EV levels from astrocytes in a dose-dependent manner (Fig. 6b and c). The reduction of EV biogenesis from astrocytes upon GW4869 treatment was also confirmed through Western blot. GW4869 reduced the levels of Flotillin-2 and Alix in the EV lysates in a dose-dependent manner (Fig. 6d). GW4869 treatment markedly decreased ZIK RNA in infected astrocytes, suggesting that GW4869 inhibits ZIKV infection in astrocytes (Fig. 6e). To confirm GW4869 inhibition of ZIKV infection, we used ICC to detect viral antigens in infected astrocytes. Treatment with GW4869 dramatically reduced the number of ZIKV-positive astrocytes in the infected cultures (Fig. 6f–j). GW4869 treatment also dramatically decreased ZIKV RNA in the supernatants and reduced viral plaque numbers in PFA (Fig. 6k–m). Similarly, treatment with GW4869 significantly decreased EV numbers in ZIKV MR766 strain-infected astrocytes (Fig. 7a and b). GW4869 reduced the levels of Flotillin-2 and tTG (Tissue transglutaminase, another EV marker) in EV lysates (Fig. 7c), suggesting that GW4869 effectively reduces EVs in infected astrocytes. GW4869 treatment markedly decreased ZIKV RNA in infected astrocytes (Fig. 7d) and in supernatants (Fig. 7e). GW4869 treatment also dramatically decreased viral plaque numbers in PFA (Fig. 7f). Together, these data suggest that GW4869 is an effective inhibitor for ZIKV infection in astrocytes.

To determine the mechanism of GW4869 inhibition of ZIKV infection, we used a viral attachment assay as previously described^50^ that quantified viral RNA upon initial exposure to the virus. Astrocytes were treated with GW4869 and then infected with ZIKV for 2 h at 4 °C. Quantification of viral RNA revealed that trypsin digestion, which removed all attached virions, did significantly reduce ZIKV RNA. In contrast, GW4869 treatment did not significantly reduce the ZIKV RNA levels in either MR766- (Supplementary Figure S8a and b) or PRVABC59-infected astrocytes (Supplementary Figure S8a and c), suggesting that GW4869 does not target ZIKV attachment in astrocytes. AXL has been proposed as a cell surface receptor critical for ZIKV infection^14^. We tested whether GW4869 decreases AXL, thus dampening the infection in astrocytes. However, consistent with the viral attachment data, treatment with GW4869 did not significantly change AXL expression in astrocytes (Supplementary Figure S9a and b), suggesting that the mechanism of GW4869-induced viral suppression is not likely through regulation of AXL. We also performed GW4869 treatment post ZIKV infection (Supplementary Figure S10a). At 24-h post ZIKV infection, we treated astrocytes with GW4869 and found that GW4869 was effective in reducing the release of Zika virions (Supplementary Figure S10b), as well as in reducing intracellular (Supplementary Figure S10c) and extracellular ZIKV RNA levels (Supplementary Figure S10d). Therefore, treatment with GW4869 post ZIKV infection is also effective in suppressing Zika virus propagation and release.

## Discussion

Astrocytes make up about one-half of a person’s brain cells and are increasingly recognized as a critical cell type for both the proper development and health of the CNS. In this manuscript, we use a unique primary human fetal astrocyte culture and demonstrate productive and specific ZIKV infection in this cell type. Interestingly, infection is associated with a significant higher number of EVs than those of mock-infected controls. Inhibition of nSMase2 through a cell-permeable inhibitor GW4869 decreases EV levels, suppress ZIKV RNA, and reduce Zika virion release in astrocytes, implicating the impact of EVs and the upstream ceramide pathway in ZIKV replication and likely its pathogenesis. Therefore, strategies targeting the EV upstream ceramide pathway may become a novel therapeutic approach to halt ZIKV infection.

The implication of ZIKV infection in human fetal astrocytes has a significant clinical relevance to ZIKV-associated microcephaly. Although MR766 strain of ZIKV infects human NSCs and leads to NSC cell death^31–33^, it is not known whether other cell types in the CNS participate in ZIKV neuropathogenesis. Our data suggests that ZIKV specifically and efficiently infects human fetal astrocytes and leads to a productive infection state with cytopathic effects. The data are consistent with recent investigations on ZIKV infection in primary human neural progenitors that exhibited cytopathic effects^51^. The data are also corroborated with recent studies in microcephalic brains showing that Zika viral antigens were present in neurons and glia cells^9^. Furthermore, in a recent mouse study, blood-borne ZIKV administration led to infection of adult NSCs and S100b-expressing astrocytes in the brains^52^. Because astrocytes have diverse regulatory and essential supporting roles in the developing and adult CNS^53^, they may actively participate in ZIKV neuropathogenesis by either serving as an important viral transmission cell type or changing its essential regulatory roles.

Astrocytes arise from neuroepithelial progenitor cells in the embryonic forebrain and neural tube^54^. At around embryonic day (E) 9 in mice, neuroepithelial progenitor cells give rise to radial glia, which are the primary progenitor cells for both neurons and astrocytes during embryogenesis. In human, ZIKV infection is of a particularly concern for maternal infection during the first trimester of pregnancy. Detection of ZIKV viral RNA and antigens in brain tissues of cases with congenital Zika infection and placental tissues of early abortions has provided a direct link between ZIKV infection and microcephaly. The astrocytes used in the current study are derived from brain tissues of aborted fetus of a gestational age ranging between 12 and 16 weeks. The ZIKV infection of these astrocytes appear to be quite specific since both TuJ1-positive neurons in the astrocyte cultures and SOX2-positive NPCs derived from the fetal brains appear to be less susceptible compared with astrocytes. The susceptibility of human astrocytes to ZIKV is in agreement with a recent publication that used a human cortical tissue slice culture^55^. Similar findings on radial glia cells have also been reported in cultured primary human brain cells^51^ and primary human organotypic brain slice culture^56^. However, our NPCs data differ from those of radial glia and human pluripotent stem cell-derived NPCs in term of ZIKV infection. The prior publications found that radial glia and human pluripotent stem cell-derived NPCs are susceptible to ZIKV infection^16, 31, 55^, whereas our NPCs are less susceptible to ZIKV infection compared with astrocytes. Despite that both of these cell types express GFAP and SOX2, it is possible that radial glia and human pluripotent stem cell-derived NPCs are more primitive, and our NPCs are in a different differentiation stage. This possibility is supported by our gene analysis that finds lower but comparable levels of glutamate transporters EAAT1/GLAST and EAAT2 in NPCs compared with astrocytes. Currently, there is still a lack of definitive markers for astrocyte differentiation and maturation from radial glia and NPCs. Future studies should use those markers to determine whether cells of different astrocyte maturation stages have differential susceptibilities to ZIKV infection.

Our data suggest GFAP-negative cells could be infected with ZIKV. GFAP is currently used as a routine antigenic marker for normal developing, mature, and activated astrocytes. This protein is typically absent in primitive or neoplastic neuroepithelial cells, oligodendrocytes, vascular endothelium, meningeal cells, and fibroblasts. The nature of those GFAP-negative cells remains unclear in our studies. Our data suggest that they are neither NPC nor microglia. They are also unlikely to be neurons since TuJ1-positive neurons are rarely infected. Since staining with S100B reveals that ZIKV infects astrocytes with different maturation stages, it is likely that those GFAP-negative cells are still astrocytes.

Our study also reveals a possible correlation between ZIKV infection-associated microcephaly and EVs. Viral infection including HIV-1 is known to augment exosomal pathways and viral proteins are found within EVs (see a recent review^28^). Using NanoSight and Western blot, we demonstrated a significant increase of EV biogenesis upon ZIKV infection. EV characterizations through TEM, Western blots for EV markers, and NanoSight for EV size/concentrations are well established. Our characterization found little evidence of apoptotic bodies in the EVs since the size of EVs was overwhelmingly smaller than 300 nm, whereas typical apoptotic bodies are more than 500 nm. In addition, EV isolation may contain virions and Zika virions may be mistaken as EVs during data interpretation. Mature Zika virions are around 50 nm in diameter^10^, which appear to be in the lower end of the NanoSight detection. Based on the data from PFA and NanoSight, EV number (10^8^–10^10^/ml) greatly exceeds the number of viral particles (10^4^–10^6^/ml) in the supernatant. Therefore, we conclude that the particles detected by the NanoSight are predominantly EVs, including exosomes and microvesicles.

Our current studies demonstrated that inhibition of nSMase2 by GW4869 in astrocytes is effective in suppressing ZIKV infection. Ceramide and sphingomyelin, both of which are products of nSMase2, are essential lipids for EV. However, exactly how this ceramide pathway is exploited to support viral infection remains unclear. The ceramide pathway may facilitate viral infection by enhancing viral RNA and protein delivery through EVs^29^. There are six structural proteins and seven non-structural proteins encoded by ZIKV genome and several of these proteins have been identified as cytopathic factors^57, 58^. It is possible that one or more of these proteins are present in the EVs that subsequently induce cytopathic effects or enhance virulence of ZIKV. Specific lipid classes, including fatty acid, phosphatidylethanolamine, and sphingolipids, have been implicated in other family members of flavivirus infections^59^. Investigations on the role of specific lipids in ZIKV infection will shed light on the identification of novel therapeutic targets in the fight against the current ZIKV epidemic.

In summary, we describe productive ZIKV infection in a unique primary human fetal astrocyte culture. Investigation on ZIKV infection in human astrocytes has revealed an increase of EV levels after ZIKV infection. Targeting nSMase2 with its specific inhibitor GW4869 is able to suppress ZIKV propagation and reduce virion release. The study contributes to new knowledge of ZIKV neuropathogenesis through characterization of ZIKV infection in fetal astrocytes. Furthermore, the identification that nSMase2 suppression can effectively halt ZIKV propagation and release might lead to a novel therapeutic strategy.

## Materials and methods

### Ethics statement

All experiments for human fetal astrocyte generation were performed with the approval of the Scientific Research Oversight Committee at the University of Nebraska Medical Center (UNMC). Human fetal brain tissues were obtained from elective aborted specimens (gestational age 12–16 weeks) following completion of the abortion procedure through collaborative works with the Birth Defects Research laboratory at the University of Washington. The protocol is in compliance with all relevant state and federal regulations and is approved by the University of Washington Institutional Review Board (IRB, approval number: 96-1826-A07) and UNMC IRB (Approval number: 123-02-FB). Informed consent was obtained with all subjects using an IRB-approved consent form at the University of Washington. All consenting subjects were donors of fetal tissue that were 19 years of age or older with clear comprehension. The UNMC investigators do not have access to signed consent forms.

### Preparation of ZIKV viral stock

Experiments involving live Zika virus or viral stocks were performed exclusively inside a BioSafety Level 2+ laboratory. All procedures utilized in this study were approved by the Institutional Biosafety Committee (IBC 16-05-013BL2) and followed biosafety level II practices as shown in the National Institutes of Health (NIH) Guideline Appendix G-II-B. ZIKV strains MR766 and PRVABC59 were purchased through ZeptoMetrix Corp., Buffalo, NY, and propagated in Vero cells. Vero cells were originally from ATCC (CCL-81) and were a gift from Dr. Kaihong Su (UNMC) and were maintained in Dulbecco’s Modified Eagle Medium (DMEM) with 5% fetal bovine serum (FBS). Mock- or ZIKV-infected Vero cells were further incubated with DMEM supplemented with 5% FBS. At 48 h post infection, cells were switched to serum free media and incubated for 24 h. Conditioned media from mock- and ZIKV-infected Vero cells were harvested, centrifuged at 300 × *g*, and stored at −80 °C as the mock-infected control and viral stock, respectively. ZIKV titers were determined the PFU per milliliter through PFA.

### ZIKV PFA

Vero cells were plated into 12-well plates at 5 × 10^5^ cells/well the day before infection. On the day of infection, the monolayers of Vero cells were inoculated with 100 μl of 10-fold serial dilutions of viral stocks and incubated at 37 °C for 1 h. After viral inoculation, medium containing ZIKV particles was removed and 1 ml overlay containing 0.6% molecular biology grade agarose (Agarose Unlimited, Alachua, FL) in Modified Eagle Medium (Gibco) with 2% FBS. Cells were maintained at 37 °C in 5% CO2 for 4 days. On day 5, cells were fixed with 4% paraformaldehyde solution in PBS and stained with 1% crystal violet solution in 20% methanol in water. Viral plaques were photographed using a CanonScan 9950F scanner and each plaque was counted as a PFU. Viral titer was calculated as PFU/[volume virus (ml) × (dilution factor)].

### Human fetal astrocyte and NPC cultures

Human fetal astrocytes were derived from the single-cell isolation process of fetal brain tissues previously described^60, 61^. Briefly, dissociated brain tissue was incubated with 0.25% trypsin for 30 min, followed by neutralization with 10% FBS, and further dissociated by trituration. The single-cell suspension was cultured at a density of 2 × 10^7^ cells/150 cm^2^ in DMEM/F12 (Thermo Fisher Scientific, Waltham, MA), supplemented with 10% FBS, and an antibiotic mixture containing penicillin, streptomycin, and neomycin (Thermo Fisher Scientific). The adherent astrocytes were treated with 0.25% trypsin after 2 weeks in culture and the cell suspension was cultured under the same conditions to enhance purity. Astrocyte preparations were assessed by immunocytochemical staining using antibodies to glial fibrillary acidic protein (GFAP, Dako Corp., Carpinteria, CA). This process yields a culture of >95% pure astrocytes. Human NPCs were isolated from fetal brain tissues as previously describe^39–41^, cultured in substrate-free tissue culture flasks, and grown as neurospheres in NeuroCult NSC basal medium with proliferation supplement (STEMCELL Technologies), 10 ng/mL recombinant human basic fibroblast growth factor (Sigma-Aldrich), and 20 ng/mL recombinant human epidermal growth factor (R&D Systems). For ZIKV infection, NPCs were seeded on poly-d-lysine-coated coverslips at the density of 0.1 million/well for two days before the infection. Astrocytes and NPCs of low passage number (passage number < 6) were used for the current study. The culture and infection protocols of human fetal astrocytes and NPCs had appropriate approvals from the UNMC Institutional Biosafety Committee and Institutional Review Board.

### Isolation of EVs

EVs were isolated from the supernatants of mock-infected and ZIKV-infected astrocytes through differential centrifugations with or without nSMase2 inhibitor GW4869 (Sigma-Aldrich) at different dosages as previously described^47^. Briefly, one day before EV isolation, cultures were switched to fresh astrocyte culture medium to exclude residual virions during the initial infection from final EV isolation and analysis. For EV isolation, culture supernatants were first centrifuged at 300 × *g* for 10 min to remove free cells, at 3000 × *g* for 20 min to remove cellular debris, and then 10,000 × *g* for 30 min to remove free organelles. Lastly, EVs were collected by ultracentrifugation at 100,000 × *g* for 70 min at 4 °C. To prepare EVs for Western blotting, the EVs pellets were lysed in M-PER mammalian protein extraction reagent (Thermo Fisher Scientific).

### Negative staining and TEM

EVs were fixed with 2% glutaraldehyde and 2% paraformaldehyde solution and then spread on the silicon monoxide and nitro-cellular film coated copper grid. The droplets were removed with filter paper, air-dried at room temperature, and incubated with Nanovan negative stain. After air-dried for 2 min, the grid was subjected TEM (FEI Tecnai G2 Spirit TWIN).

### Western blot

Protein concentrations were determined by Bradford protein assay (Thermo Fisher Scientific). Proteins in WCL and EVs lysates were separated through SDS PAGE and electrophoretically transferred to polyvinyldifluoridene membranes (Millipore, Billerica, MA and Bio-Rad, Hercules, CA). Membranes were incubated overnight at 4 °C with polyclonal antibodies for flotillin-2 (Cell Signaling Technology, Danvers, MA), Alix (Santa Cruz Biotechnology, Dallas, TX), AXL (Cell Signaling Technology), ZIKV envelop protein (GeneTex, Inc. Irvine, CA), as well as β-actin and GFAP (Sigma-Aldrich) followed by horseradish peroxidase-linked secondary anti-rabbit or anti-mouse secondary antibodies (Cell Signaling Technology). Antigen-antibody complexes were visualized by Pierce ECL Western Blotting Substrate. For quantification of the data, films were scanned with a CanonScan 9950F scanner and images were analyzed using the public domain NIH image program (developed at the U.S. National Institutes of Health and available on the internet at http://rsb.info.nih.gov/nih-image/).

### Nano-particle tracking analysis

A NanoSight NS 300 (Malvern Instruments Inc., Westborough, MA) equipped with an sCMOS camera was utilized to analyze the size distribution and concentration of EVs. NanoSight applied NTA, which is a combination of light scattering and Brownian motion technology, to measure the size distribution and concentration of EVs in supernatants. After EV isolation, the pellets were first resuspended in 80 μl of filtered PBS and then diluted by 100 times. The conditions of the measurements include the temperature of 25 °C, viscosity of 1 cP, 25 s per capture frame, and a measurement time of 60 s. All of the conditions remained the same amongst all of the samples.

### Quantitative real-time RT-PCR

Total mRNA was isolated with TRIzol Reagent (Thermo Fisher Scientific) and RNeasy Mini Kit (QIAGEN Inc., Valencia, CA) using the manufacturer’s recommendations. The reverse transcription was performed using Verso cDNA synthesis Kit (Thermo Fisher Scientific). The RT-PCR analyses of ZIKV RNA were performed using SYBR. Select Master Mix (Thermo Fisher Scientific) with 0.5 μl of cDNA, corresponding to 1 μg of total RNA in a 15 μl final volume, 1.5 μl H_2_O, 7.5 μl SYBR Green, 5.5 μl oligonucleotide primer pairs (synthesized at Thermo Fisher Scientific) at 10 μM. Primers used for real-time RT-PCR were ZIKV RNA: forward sequence 5-TGGGAGGTTTGAAGAGGCTG-3, reverse sequence 5-TCTCAACATGGCAGCAAGATCT-3, as previously reported^31^; GAPDH: forward sequence 5-GGAGCGAGATCCCTCCAAAAT-3, reverse sequence 5-GGCTGT TGTCATACTTCTCAT GG-3. PCR program: 1, 50 °C for 2 min; 2, 95 °C for 2 min; 3, 95 °C for 15 s; 4, specific annealing temperature for 15 s; 5, 72 °C for 1 min. Steps 2–4 were repeated 40 times. All samples were amplified in triplicate for analysis. Relative ZIKV RNA levels were determined and standardized with a GAPDH internal control using comparative ∆∆CT method. For ZIKV RNA levels in GW4869 treatment, and all of the supernatants, real-time RT-PCR was carried out using the one-step quantitative TaqMan assay in a StepOne^TM^ Real-Time PCR system (Thermo Fisher Scientific). Primers used for real-time RT-PCR include ZIKV (forward sequence: 5-TTGGTCATGATACTGCTGATTGC-3, reverse sequence: 5-CCTTCCACAAAGTCCCTATTGC-3, and probe sequence: 5′-CGGCATACAGCATCAGGTGCATAGGAG-3) as previously reported^62^, 18S rRNA endogenous control (Catalog number: 4333760F, Thermo Fisher Scientific) and GAPDH (Catalog number: 4310884E, Thermo Fisher Scientific). Relative ZIKV mRNA levels were determined and standardized with a GAPDH or 18S rRNA endogenous control using comparative ∆∆CT method. For ZIKV in supernatants, since there was not endogenous control, an equal volume of extracted RNA was used in real-time RT-PCR. For selected experiments, quantitative genomic RNA from ZIKV (NR-1838DQ, ATCC) was used as a standard to calculate viral copies. All primers used in the study were tested for amplification efficiencies and the results were similar.

### Immunocytochemistry

The cultured cells were fixed in 4% paraformaldehyde for 20 min at room temperature and then incubated with methanol for 20 min at −20 °C. Fixed cells were blocked with 3% bovine serum albumin in PBS and then incubated with primary antibodies to Flavivirus Group Antigen (clone D1-4G2-4-15, Millipore, Billerica, MA), GFAP (Dako), or cleavage caspase 3 (Cell signaling Technology) overnight. At the second day, the cells were washed with PBS for three times and incubated for 1 h at room temperature with the secondary anti-mouse IgG antibody (coupled with green dye, Alexa Flour 488, Molecular Probes, Eugene, Oregon). Nuclear DNA were labeled with 4′,6-diamidino-2-phenylindole (DAPI; Sigma-Aldrich, St. Louis, MO) for 10 min after the secondary antibody at room temperature. Cover slips were mounted on glass slides with mounting medium (Sigma-Aldrich). Fluorescent images were obtained using a Zeiss 710 Confocal Laser Scanning Microscope (Carl Zeiss, Oberkochen, Germany). All obtained images were imported into Image-ProPlus, version 7.0 (Media Cybernetics, Sliver Spring, MD) to quantify the number of infected cells. The assessors were blinded during image acquisition or quantification.

### Cell viability assay

Cell viability was determined by a colorimetric CellTiter 96^®^ AQueous One Solution Assay (Promega, Madison, WI) based on the manufacture’s instruction. Assays were performed by adding a small amount of the CellTiter 96^®^ AQueous One Solution Reagent, which contained a tetrazolium compound [3-(4,5-dimethylthiazol-2-yl)-5-(3-carboxymethoxyphenyl)-2-(4-sulfophenyl)-2H-tetrazolium, inner salt; MTS] and an electron coupling reagent (phenazine ethosulfate; PES), directly to culture wells, incubating for 1 h, and then recording absorbance at 490 nm with a 96-well plate reader. The quantity of formazan product as measured by the amount of 490 nm absorbance is directly proportional to the number of living cells in culture.

### Statistical analysis

Data were analyzed as means±SEM unless otherwise specified. The data were evaluated statistically by the analysis of variance (ANOVA) followed by Tukey test for pairwise comparisons by using GraphPad Prism software. Significance was considered when *p* < 0.05. All in vitro experiments were performed with at least three donors to account for any donor-specific differences. The sample sizes for in vitro experiments were provided in figure legends and all assays were performed at least three times in triplicate or quadruplicate.

## Electronic supplementary material

Supplementary Information
