# Supplementary material for: Zika virus propagation and release in human fetal astrocytes can be suppressed by neutral sphingomyelinase-2 inhibitor GW4869
Source: Cell Discov. 2018 Apr 24;4:19. doi: 10.1038/s41421-018-0017-2 (PMC5913238; doi:10.1038/s41421-018-0017-2)
Supplement: Supplementary file 1 — Supplementary Information [file 41421_2018_17_MOESM1_ESM.pdf]

Supplemental Figure 1.

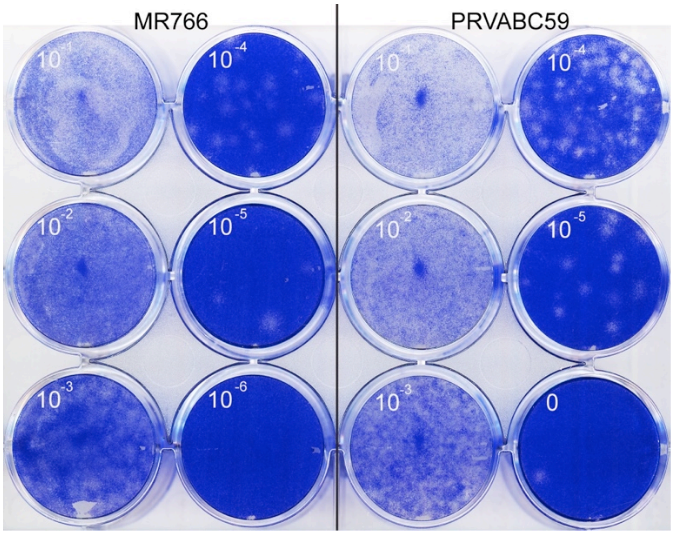

Supplemental Figure 1. Quantification of ZIKV through viral plaque assays. Viral titers in the cell-free viral stocks were determined by PFA at 4-day post inoculation. Viral plaques were visualized through crystal violet staining and representative pictures of the viral strains were shown.

Supplemental Figure 2.

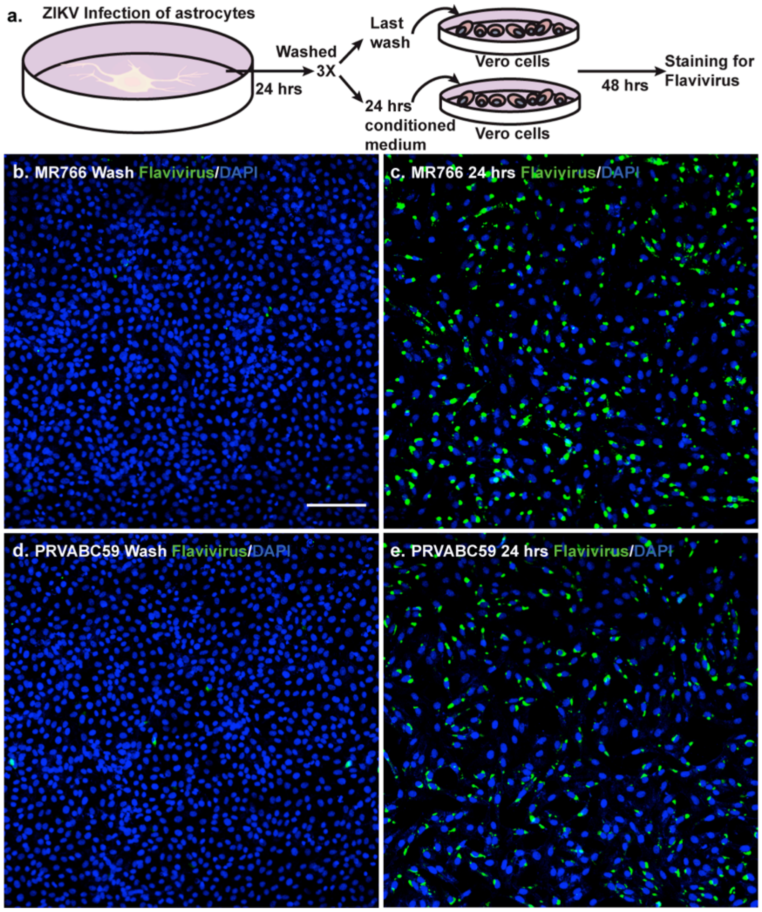

Supplemental Figure 2. ZIKV infection of astrocytes is a productive infection. Astrocytes were infected with ZIKV for 24 hours. The culture was washed and the last wash with PBS, as well as supernatants from another 24-hour incubation in the infected cultures, were collected as conditioned medium and added to Vero cells. Flavivirus antigens were confirmed through ICC at 48 hours post incubation with supernatants or last-wash PBS. **a**) A schematic diagram of the experimental process is shown. **b-e**) At the experimental endpoint, ICC of flavivirus antigen (green color) was performed. DAPI (blue color) was used as a nuclear counterstain. Scale bar: 100 µm. Panels are representative of three separate donors. Images were acquired through a Zeiss LSM 710 confocal microscope.

Supplemental Figure 3.

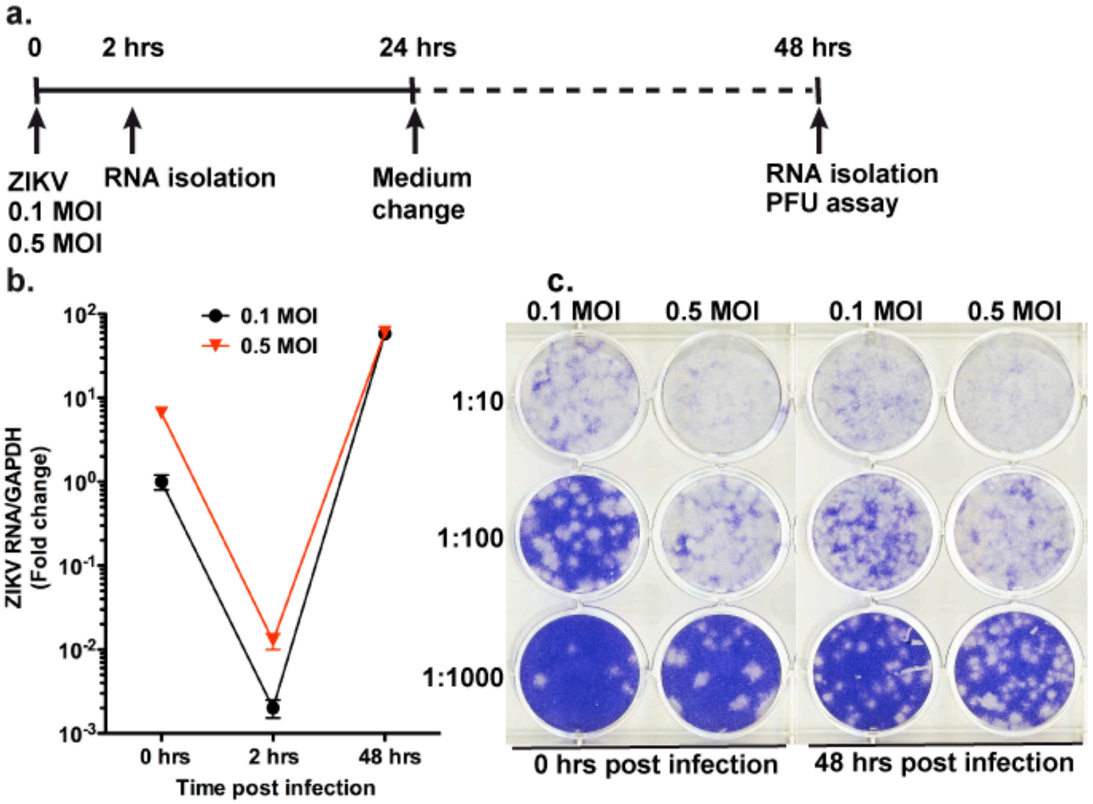

Supplemental Figure 3. ZIKV replication and virion production in primary fetal astrocytes.

a) Primary human fetal astrocytes were infected with ZIKV at 0.1 or 0.5 MOI for 2 hours or 48 hours and ZIKV RNA and virions were quantified. **b**) RNA was isolated from supernatant during infection (0 hr) or from astrocytes at 2 and 24 hours post infection. Expression of ZIKV RNA was determined through real-time RT-

PCR and data were normalized to the ZIKV RNA levels during infection. **c**) Number of ZIKV virions was determined by PFA at 0- and 48-hour post infection.

Supplemental Figure 4.

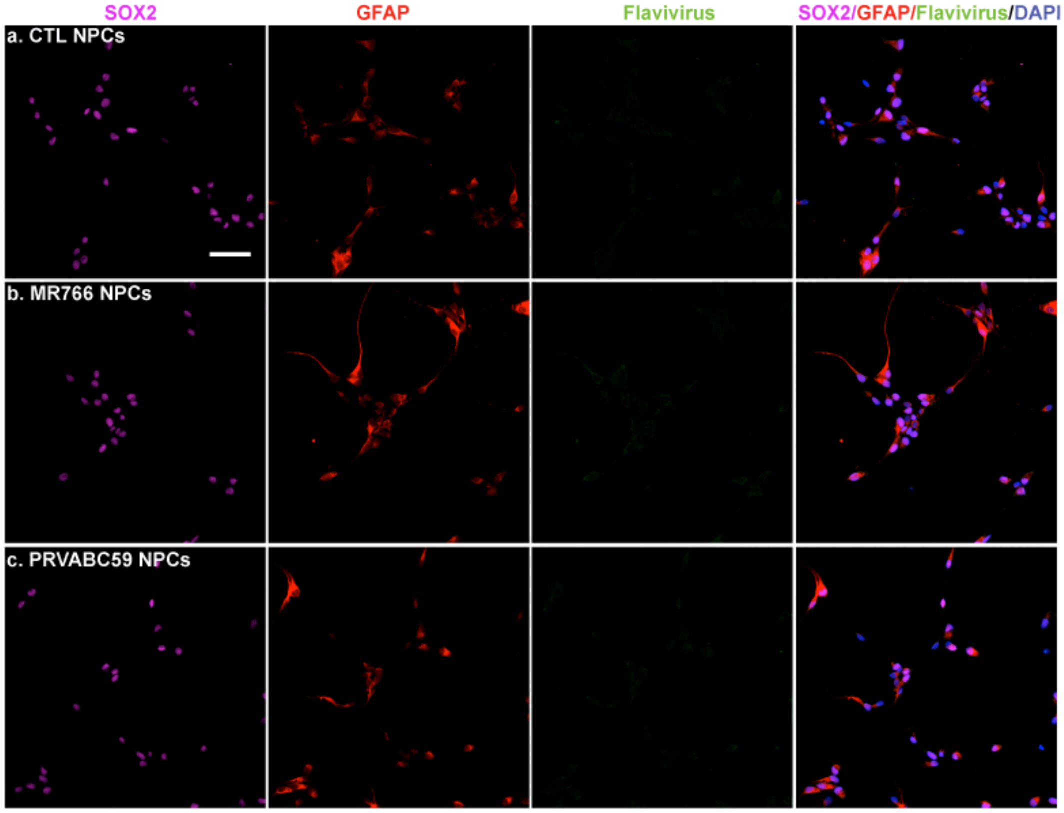

Supplemental Figure 4. Human NPCs are less susceptible to ZIKV infection than astrocytes. Human NPCs were mock-infected or infected with ZIKV strains MR766 or PRVABC59 at the MOI of 0.5 for 24 hours, washed, then cultured for another 24 hours. **a-c**) At the experimental endpoint, ICC of SOX2 (purple color), GFAP (red color), and flavivirus antigen (green color) was performed. DAPI (blue color) was used as a nuclear counterstain. The ICC was performed in the same setting as those in Figure 2a-c. Panels are representative of three separate donors. Scale bar: 50 µm. CTL, control.

Supplemental Figure 5.

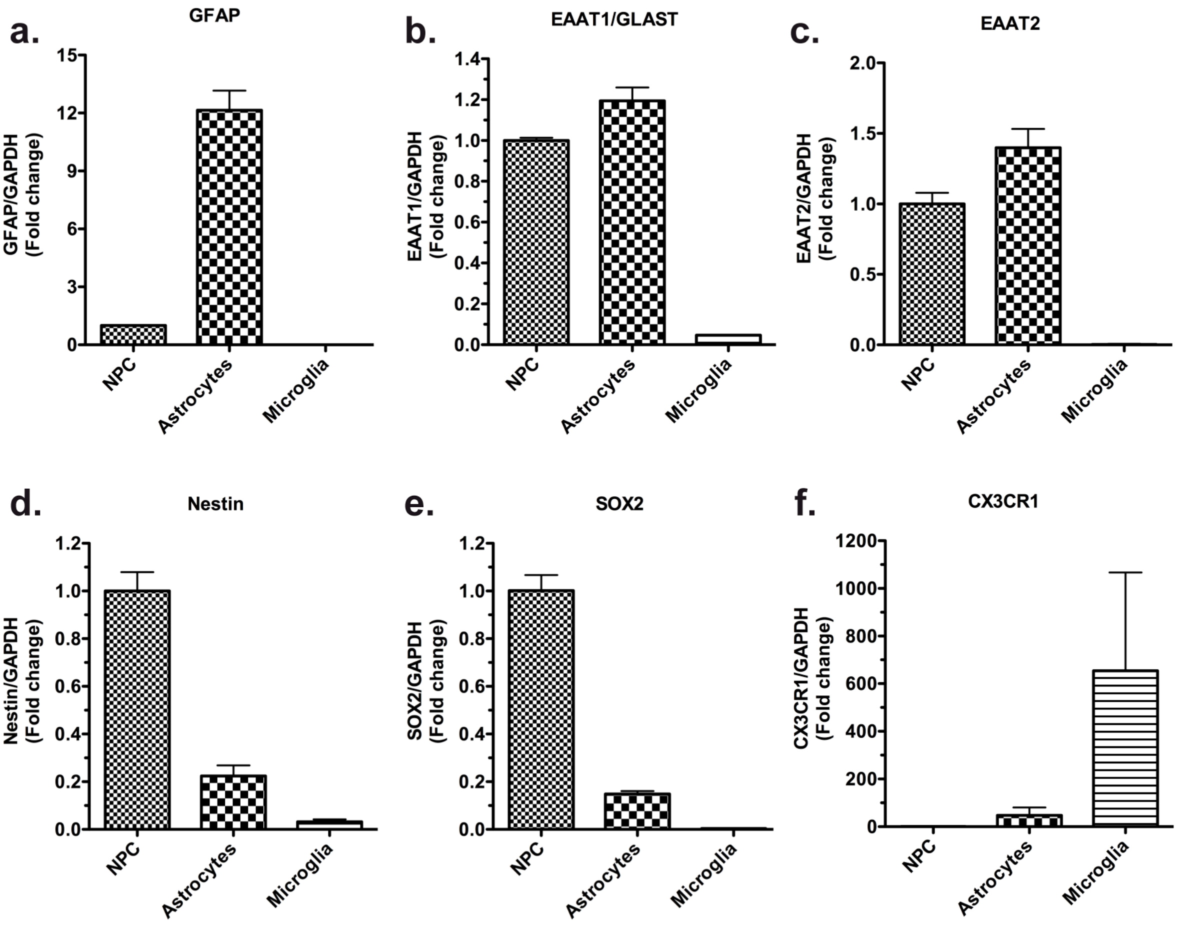

Supplemental Figure 5. Marker gene expressions of the primary astrocytes. a-f) Primary human fetal astrocytes, NPCs, and microglia were all derived from human fetal brain tissues. NPCs were maintained in the neurosphere form. At the experimental endpoint, all cells were subjected to total RNA extraction and gene expressions of GFAP (**a**), EAAT1/GLAST (**b**), EAAT2 (**c**), Nestin (**d**), SOX2 (**e**), and CX3CR1 (**f**) were determined through real time RT-PCR. Data were normalized to GAPDH and presented as fold change compared to NPC.

Supplemental Figure 6.

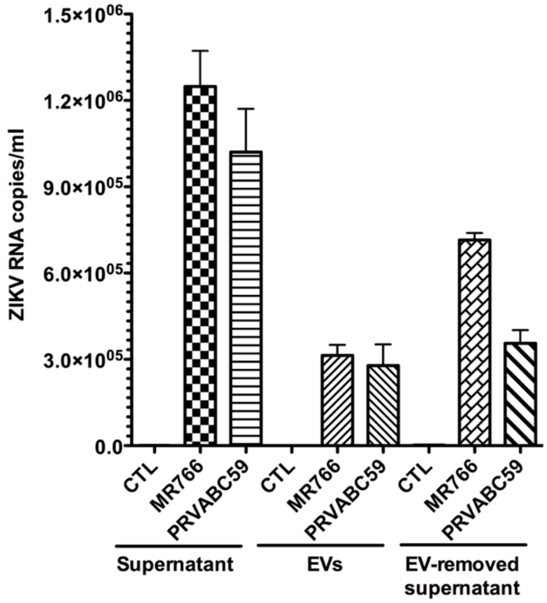

Supplemental Figure 6. Analysis of viral RNA in supernatants, EVs, and EV-removed supernatants of infected cultures. Astrocytes were infected with ZIKV stains PRVABC59 or MR766 for 24 hours and the cultures were washed and supplemented with fresh media for another 24 hours. EVs were isolated from supernatants through ultracentrifugation. ZIKA RNA was detected in total RNA isolated from an equal volume of cell-free supernatants, their derived EVs, and EV-removed supernatants through real time RT-PCR. Quantitative Genomic RNA from ZIKV (ATCC) was used as standard for viral copy determination. CTL, control.

Supplemental Figure 7.

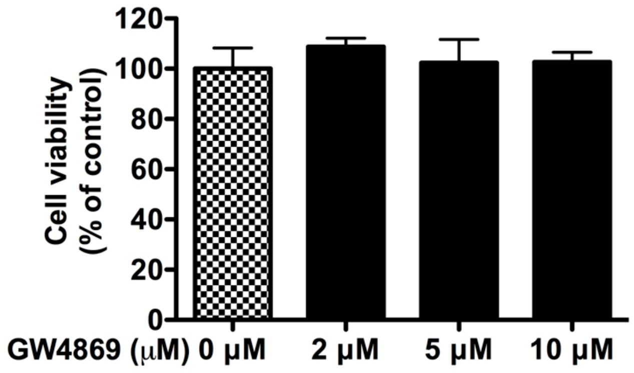

Supplemental Figure 7. GW4869 does not change the viability of ZIKV-infected astrocytes. Astrocytes were infected with ZIKV stains PRVABC59 and treated with doses of GW4869 similar with Fig. 7. At the 48-hour end point, cell viability was determined by a colorimetric MTS assay. Results were normalized as percentage of DMSO (solvent) control for GW4869.

Supplemental Figure 8.

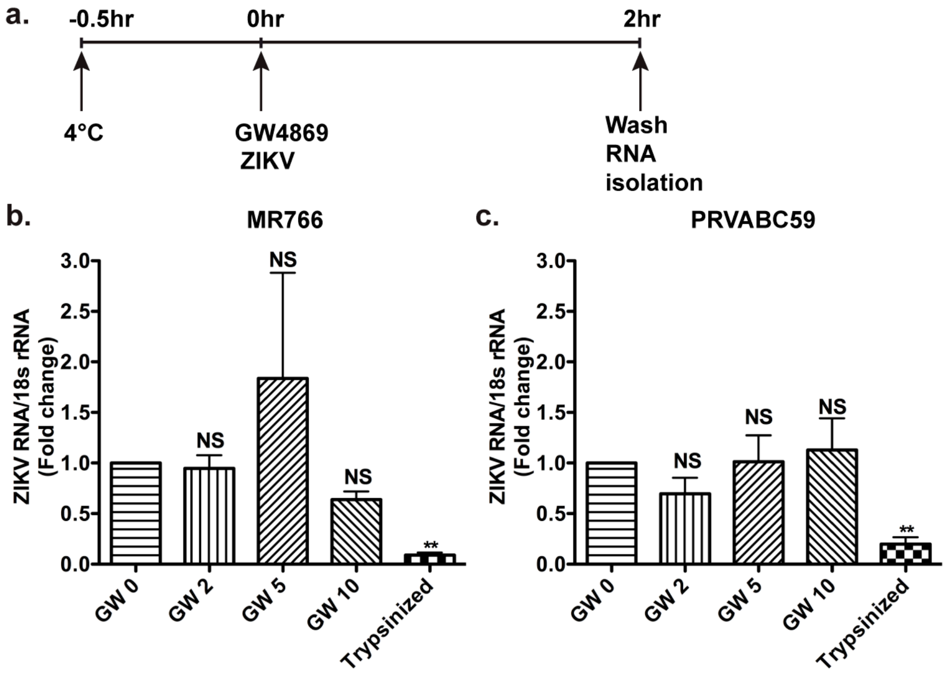

Supplemental Figure 8. GW4869 does not affect ZIKV viral attachment in astrocytes. a) Primary human fetal astrocytes were pre-incubated in 4°C for half an hour and then treated with GW4869 along with ZIKV infection. Both MR766 and PRVABC59 strains of ZIKV were used for the infection at the MOI of 2. After treatment and infection in 4°C for 2 hours, cultures were washed with fresh medium for three times and RNA

was isolated from whole cells. **b-c**) Expression of intracellular ZIKV RNA was determined through real-time RT-PCR. Data were normalized to GAPDH and presented as fold change compared to DMSO (solvent) control for GW4869. One experimental group was subjected to trypsin digestion to remove any attached virions. This group served as positive control for this viral attachment experiment. ** denotes $p < 0.01$; NS denotes non-significant, as compared to the DMSO control (GW0 group).

Supplemental Figure 9.

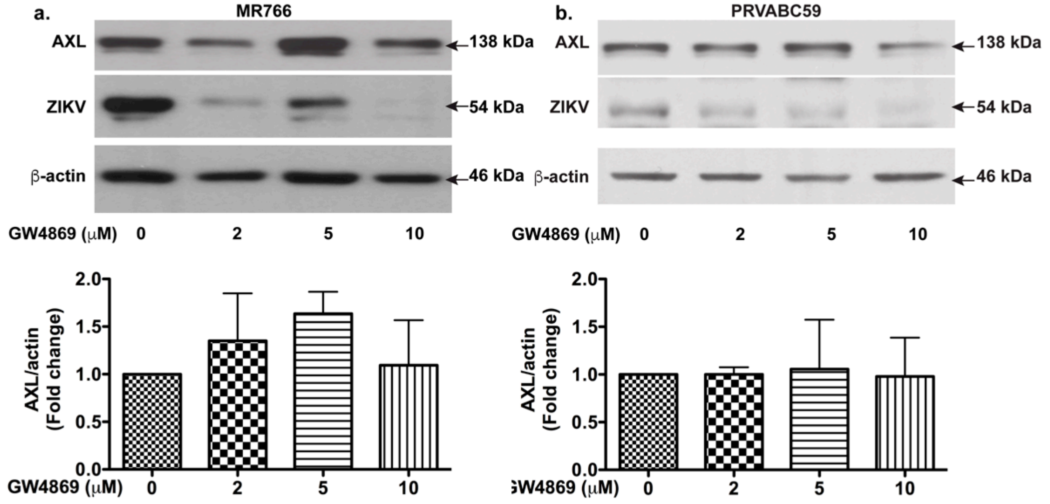

Supplemental Figure 9. GW4869 does not affect AXL expression in ZIKV-infected astrocytes. Primary human fetal astrocytes were infected with ZIKV strains MR766 (**a**) or PRVABC59 (**b**) at the MOI of 0.5 for 24 hours before GW4869 treatment. At 24 hours post GW4869 treatment, whole cell lysates were collected and the levels of AXL, ZIKV envelop protein, and β-actin were determined by Western blot. Densitometric quantifications of AXL and ZIKV envelop protein were presented as a ratio to β-actin and normalized as fold changes to that control.

Supplemental Figure 10.

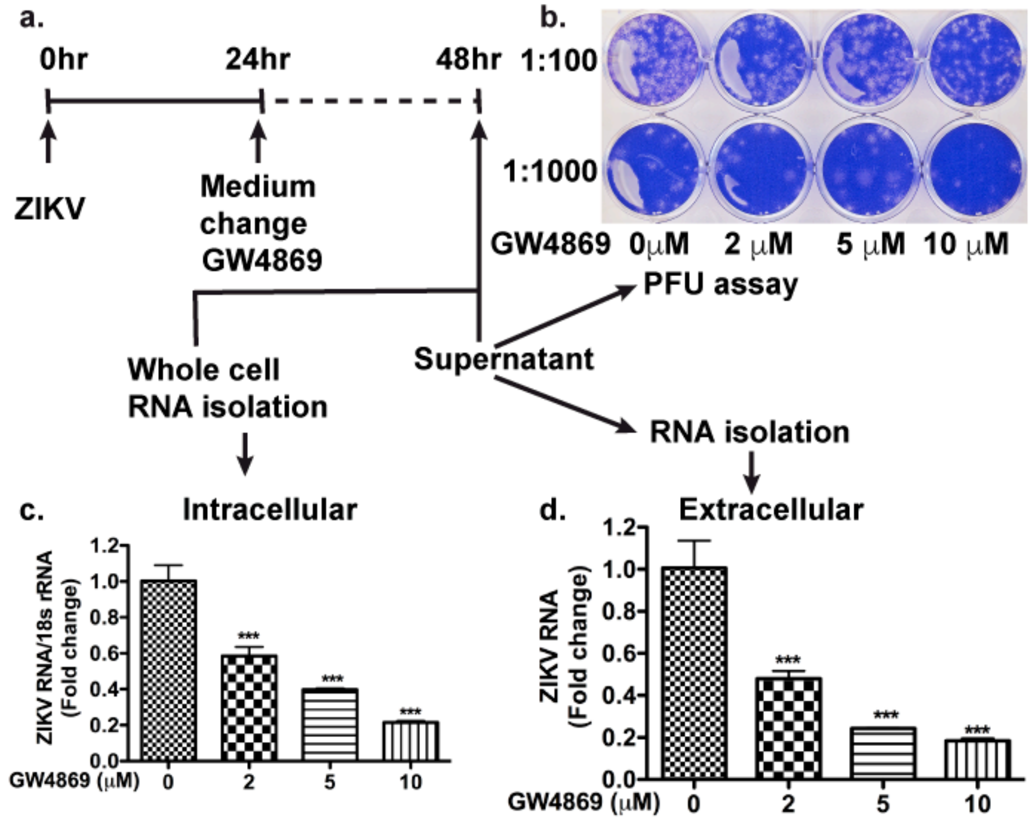

Supplemental Figure 10. GW4869 treatment post ZIKV infection inhibits viral replication in astrocytes. Primary human fetal astrocytes were infected with ZIKV at the MOI of 0.5 for 24 hours before GW4869 treatment. **a**) Experimental scheme. **b-d**) At 24 hours post GW4869 treatment, cell free supernatants were collected and subjected to PFA for determination of viral titer (**b**). RNA was isolated and expression of intracellular (**c**) and extracellular (**d**) ZIKV RNA was determined through real-time RT-PCR. Data were normalized to 18S rRNA and presented as fold change compared to DMSO (solvent) control for GW4869. *** denotes p < 0. 001, as compared to the DMSO control.
